# Metastatic Melanoma presenting 24 years after surgical resection: a case report and review of the literature

**DOI:** 10.1186/1757-1626-2-189

**Published:** 2009-11-10

**Authors:** Eoin Slattery, Diarmuid O'Donoghue

**Affiliations:** 1 Centre for Colorectal Disease, St. Vincent's University Hospital, Dublin 4, Ireland

## Abstract

**Introduction:**

Malignant Melanoma is becoming increasingly common. Recurrence is common in, with late recurrence up to 10 years being recognised. We present a case of recurrent metastatic melanoma 24 years after initial presentation, which is the longest interval reported to date.

**Case presentation:**

EF presented with iron-deficiency anaemia, lethargy, and weight loss. He had an enucleation of his left eye 24 years previously for a uveal melanoma. Endoscopy and biopsy confirmed recurrent duodenal and gastric metastasis. A staging CT demonstrated wide spread thoracic, liver, adrenal and bone metastasis. He was treated with palliative chemotherapy, and died 3 months later.

**Conclusion:**

Late presentation of metastatic melanoma is common, and should be remembered in patients with a distant history of melanoma. Even, as in our case, if the history is more than two decades previously. Treatment options are poor; earlier recognition may lead to improved survival.

## Introduction

Malignant melanoma is a neoplasm of the melanocyte. Its annual incidence has increased dramatically in recent years. It may develop in pre-existing lesions or can occur de novo. Similarly, it can present in sun-exposed areas (acute, intense sunburns are a definite risk factor) or in unexposed areas (e.g. sole of foot, eye, etc.). It accounts for 5% of all skin cancers, but causes 3 times more deaths than other skin cancers.

Early diagnosis is crucial, as metastatic or advanced disease is associated with poorer prognosis. The commonest site of presentation for men tends to be the trunk, and for women is the lower limb [1].

Surgery is the treatment of choice for localised cutaneous melanoma. Features that effect prognosis adversely are tumour thickness in millimetres (i.e. Breslow's depth), depth related to skin structures (i.e. Clark level), ulceration and presence of invasion. Adjunctive therapy, specifically Interferon or Dacarbazine, may be of benefit in patients with more advanced disease.

Recurrence of melanoma is common, even many years after the initial diagnosis. We present the case of a man presenting 24 years after his initial diagnosis, who underwent "curative" treatment at the time.

## Case presentation

EF is a 72 year old man who presented to our emergency department in February 2009. He described a 4 month history of fatigue and weight loss, with a 4 kg weight loss in the preceding 8 weeks. He had noticed a change in bowel habit, becoming constipated, over the last 3 weeks. Along with this history; he also complained of a dry, non-productive cough in the preceding two months.

He had a long-standing history of Hypertension and Hypercholesterolaemia; for which he was prescribed atorvastatin, perindopril and bendrofluazide.

He gave a history of a vision loss in his left eye due to a "tumour", which required an enucleation of his left eye 24 years previously.

His routine laboratory investigations revealed a hypochromic, microcytic anaemia (Haemoglobin 9.0 g/dL (range 13-17 g/dL), Mean Cell Volume 74fl (range 80-100fl), Mean Cell Haemoglobin 23.8 pg (range 27-32 pg)). His Erythrocyte Sedimentation Rate was elevated at 68 mm/hr (range 0-30 mm/hr). His renal and liver profile was normal, although his albumin was noted to be low at 30 g/L (range 35-50 g/L). His Chest X-ray on admission suggested hilar lymphadenopathy. His Faecal Occult Blood Test was positive.

**Figure 1:**
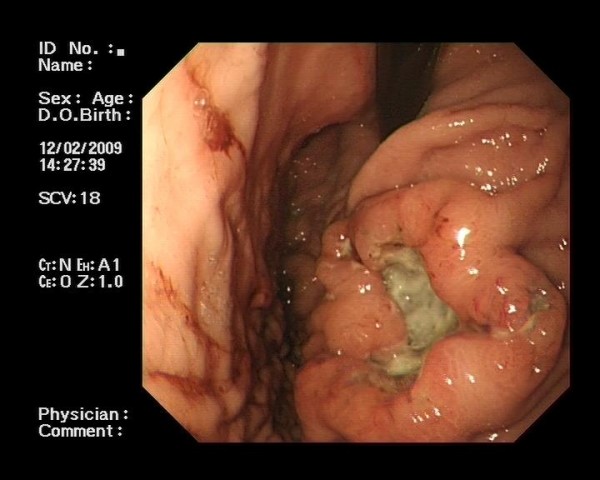
**Gastric Ulcer: Endoscopic picture of gastric metastatic melanoma**.

**Figure 2:**
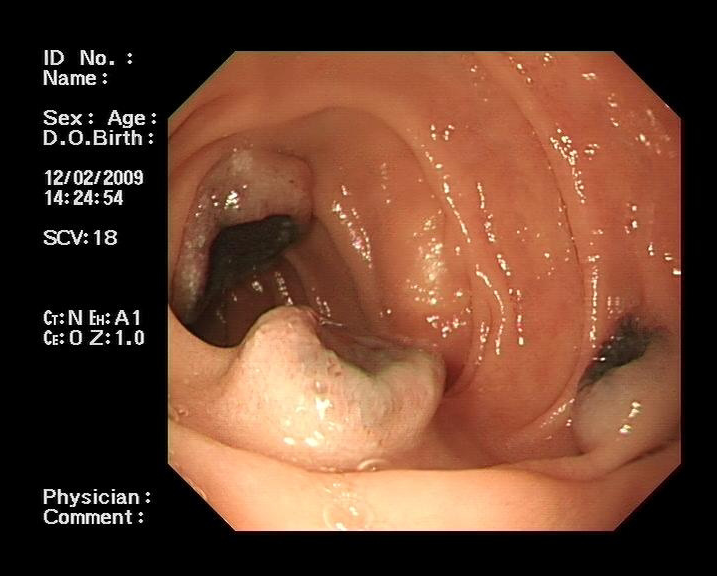
**Duodenal Ulcers: Endoscopic picture of duodenal metastatic melanoma**.

He proceeded to investigation of his iron-deficiency anaemia by gastroscopy and colonoscopy. His colonoscopy was normal. His gastroscopy, however, revealed several abnormalities. He was found to have a large, irregular fungating ulcer in the fundus of his stomach (see Figure 1). This measured 6-7 cms in maximal diameter. The appearances of which were concerning for malignancy. A second smaller ulcer was noted inferior to this. Three further irregular ulcers were noted in the second part of his duodenum (see Figure 2). Their appearances were again worrisome for malignancy. These ulcers, in contrast to the gastric ulcers however, were noted to have a quite marked central pigmentation. He had multiple biopsies from stomach and duodenum taken.

Histopathological examination revealed extensive mucosal and sub-mucosal infiltration of gastric and duodenal tissue by a malignant tumour with a solid growth pattern and pigment. Immunohistochemistry stains were positive for S100 and negative for Keratin and CAM 5.2, consistent with a diagnosis of metastatic melanoma.

He subsequently underwent a staging CT of Abdomen, Thorax and Pelvis which confirmed the endoscopically diagnosed mass in his stomach. He was noted also to have abdominal and thoracic lymphadenopathy. Bilateral adrenal metastases were seen, along with several ill-defined lesions in his liver which were felt to represent further metastasis. Multiple lung metastases and a probable bone metastasis in a lumbar vertebra were also appreciated.

He was informed of his diagnosis of metastatic melanoma, and seen by the medical oncologists with a view to palliative chemotherapy. He was commenced on Temozolomide 150 mg/m^2 ^(increasing to 200 mg/m^2^) for five days and to be repeated every 4 weeks.

He was subsequently discharged and continued his chemotherapy as an out-patient. He tolerated his treatment relatively well. However, despite this his disease progressed. He subsequently died in May 2009.

## Discussion

In retrospect, this man's prior ocular tumour was found to be a primary uveal melanoma. At the time, he underwent standard treatment which was enucleation of the affected eye. This has largely been superceded recently by more non-invasive treatments such as: radiotherapy, photocoagulation, thermotherapy, photo-dynamic therapy and local resection. Efficacy rates are similar to enucleation[2], but radiotherapy obviously has the benefit of being non-invasive.

Late recurrence of melanoma is not uncommon, five and ten-year recurrence rates are 25 and 34 percent respectively[3]. Recurrence of melanoma up to 17 years after the initial diagnosis has been reported [4]. The almost two and a half decades reported here is unusual and has not previously been recognised.

Uveal Melanoma has a five year survival rate of almost 60%, which has remained stable with newer, less invasive treatments [2]. Metastases commonly present as liver (approximately 20%) or lung lesions (about 30-35%). Gastro-intestinal metastasis accounts for about 5% of clinically apparent metastasis. Surveillance has been shown to improve life-expectancy, but not mortality and so is recommended post treatment with chest X-ray and liver function tests with or without liver imaging [5]. Duration of screening is not well established.

The prognosis of metastatic melanoma is poor, and there are no effective treatments. Median survival is reported as 2-12 months. The only licensed chemotherapeutic agent for metastatic melanoma is Dacarbazine. Response rates are modest at 6-15% (and almost all are described as partial), with a response duration of only 7-8 months [6]. In view of poor response rates, it is generally accepted that using experimental or more convenient treatments is reasonable. Temozolomide has been reported to have similar response rates to Dacarbazine, but is administered orally and so is a reasonable therapeutic option [6].

## Conclusion

A diagnosis of recurrent metastatic melanoma should always be considered in patients re-presenting in whom there is a prior history of melanoma. Our case illustrates that even after more than two decades since the initial presentation, recurrence is possible. The surveillance of patients after initial presentation remains problematic, especially in the case of uveal melanoma. Specifics, surrounding duration and type of surveillance remain unresolved. However, more problematic than those is the lack of an effective treatment option. The evidence for improving life expectancy by surveillance is poor as a consequence of a lack of a viable therapeutic option [5]. A benefit is unlikely to be seen consistently unless a more effective treatment regime is found. It is unlikely, in the case presented, that prolonged surveillance would have altered the life expectancy or mortality associated with metastatic melanoma.

Recurrent metastatic malignant melanoma is a devastating disease, with a dismal prognosis. Further work is required to determine a more effective treatment option.

## Competing interests

The authors declare that they have no competing interests.

## Authors' contributions

ES performed the endoscopy, analyzed and interpreted the patient data, and wrote the manuscript. DO'D was a major contributor in writing the manuscript. All authors read and approved the final manuscript.

## Consent

Written informed consent was obtained from the next of kin for publication of this case report and accompanying images. A copy of the written consent is available for review by the Editor-in-Chief of this journal.
